# Vaccination against Lyme disease: past, present, and future

**DOI:** 10.3389/fcimb.2013.00006

**Published:** 2013-02-12

**Authors:** Monica E. Embers, Sukanya Narasimhan

**Affiliations:** ^1^Division of Bacteriology and Parasitology, Tulane National Primate Research CenterCovington, LA, USA; ^2^Section of Infectious Diseases, Department of Internal Medicine, Yale University School of MedicineNew Haven, CT, USA

**Keywords:** Lyme disease, vaccine, reservoir, vector, tick, *Ixodes scapularis*, *Borrelia burgdorferi*

## Abstract

Lyme borreliosis is a zoonotic disease caused by *Borrelia burgdorferi sensu lato* bacteria transmitted to humans and domestic animals by the bite of an *Ixodes* spp. tick (deer tick). Despite improvements in diagnostic tests and public awareness of Lyme disease, the reported cases have increased over the past decade to approximately 30,000 per year. Limitations and failed public acceptance of a human vaccine, comprised of the outer surface A (OspA) lipoprotein of *B. burgdorferi*, led to its demise, yet current research has opened doors to new strategies for protection against Lyme disease. In this review we discuss the enzootic cycle of *B. burgdorferi*, and the unique opportunities it poses to block infection or transmission at different levels. We present the correlates of protection for this infectious disease, the pros and cons of past vaccination strategies, and new paradigms for future vaccine design that would include elements of both the vector and the pathogen.

## Introduction

In the United States, during 2000–2010, a total of 251,720 confirmed cases of Lyme disease were reported to the CDC by health departments in the 50 states, the District of Columbia, and US territories; the annual count increased 101%, from 9908 cases in 1992 to 19,931 cases in 2006 and has approached 30,000 more recently. Twelve states account for 95% of cases nationally: Connecticut, Delaware, Maine, New Hampshire, New Jersey, New York, Pennsylvania, Massachusetts, Rhode Island, Virginia, Minnesota and Wisconsin. Despite increased public knowledge of Lyme disease and improvements in diagnosis, the incidence of Lyme disease in North America has not declined. In fact, evidence indicates that in Canada and Europe the number of Lyme disease cases is on the rise (Fülöp and Poggensee, 2008; Koffi et al., 2012) and may yet increase (Ogden et al., 2008; Mannelli et al., 2012). In six reporting eastern states of Germany, for example, the incidence rose from 17.8 cases per 100,000 people in 2002 to 37.3 cases per 100,000 in 2006. Across Europe and North America, the number of reported cases is probably a significant underestimate of actual cases (Henry et al., 2011; Scott et al., 2012). Adding to the public health impact, Lyme disease spirochetes have also been identified in Asia and Australia, with the actual disease incidence yet unclear (Mayne, 2011; Stanek and Reiter, 2011).

**Figure 1:**
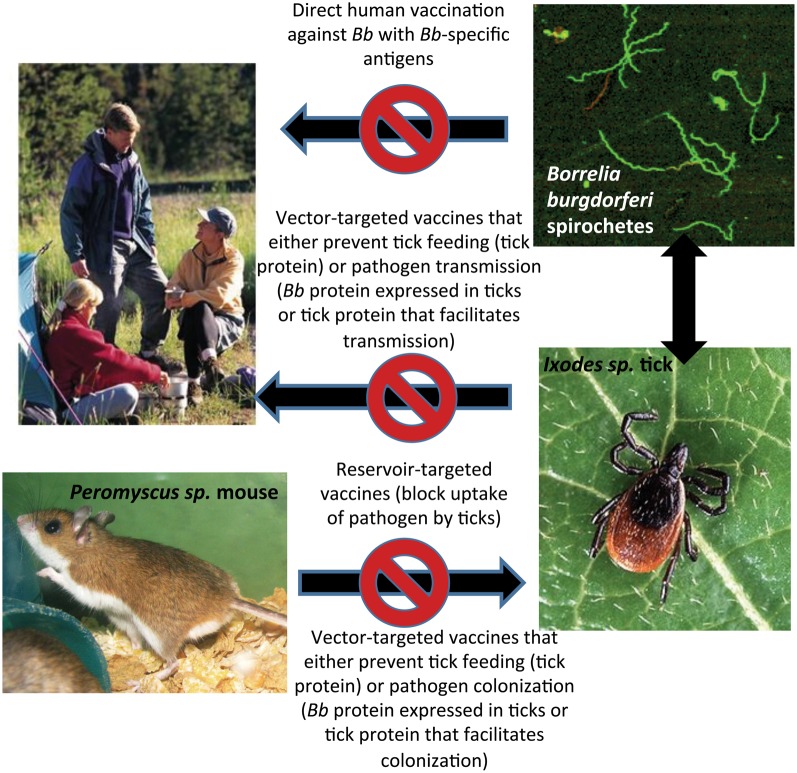
**Points at which interruption of *B. burgdorferi* transmission to humans can be achieved through vaccination**.

Vaccination against infection is a highly effective means to control the spread of disease in a population. In general, vaccines in common use protect against highly transmissible diseases and effectiveness is largely based on the generation of herd immunity. In this review, we discuss vaccination with regard to protection against Lyme disease—a disease that is not readily transmitted from person to person, but one that is both vector-borne and one whose risk is largely influenced by geography. Despite these limits of contagion, Lyme disease has become a serious and expensive public health problem. The impetus for development of a vaccine gained momentum in the 1990's and led to the approval of the first Lyme disease vaccine for human use. Only on the market for 4 years, several factors led to its failure and enthusiasm for a subsequent product may be founded more in basic science than in the pharmaceutical industry. Prominent scientists have, however, called for renewed interest in a Lyme disease vaccine (Plotkin, 2011; Poland, 2011). Among them, renowned vaccinologist Stanley Plotkin published an article calling the removal of the Lyme vaccine a “public health fiasco (Plotkin, 2011).” Notwithstanding, the possible avenues to protect against Lyme disease include interruption of transmission and infection at multiple points (Figure 1). Current research extends potential well beyond simple vaccination of humans and here we highlight specific approaches, with emphasis on vaccination against the tick vector.

## The essential antibody response

Studies in mice have shown that immunity to reinfection with *B. burgdorferi* is short-term and declines significantly by 1 year (Piesman et al., 1997). Reports of human or non-human primate infection with both the Lyme disease spirochete and Relapsing Fever-causing spirochetes also indicate that incidental hosts are likewise susceptible to reinfection (Felsenfeld and Wolf, 1975; Nowakowski et al., 1997; Golde et al., 1998). Therefore, immune responses generated during the natural course of infection are insufficient for long-term protection. Interestingly, passive immunization with serum from acute infection in mice (Barthold et al., 1997) or chronic infection in humans (Fikrig et al., 1994) has been shown to be protective against challenge in mice. In fact, the importance of antibody responses in controlling these bacterial infections is well-established (Fikrig et al., 1997; McKisic and Barthold, 2000). These and other findings indicate that the *B. burgdorferi* spirochetes alter antigen expression during infection so as to evade the antibody response and do not elicit effective memory responses to protective antigens (i.e., those that are expressed by all spirochetes and likely essential for infectivity). Thus, identification of suitable antigens for induction of protective immunity has been a challenge.

## Vaccine development: considerations

Development of protective vaccines requires appraisal of multiple factors, both common and pathogen-specific. Given the transmission mode and antigenic variation of *B. burgdorferi*, qualities that pertain specifically to this vector-borne infection must be scrutinized. As with many pathogens, the use of whole-cell lysates vs. subunit antigens is a safety concern for human use. In previous studies, cross-reactivity between anti-Borrelial whole-cell antibodies and host tissue antigens led to emphasis on a subunit vaccine (Aberer et al., 1989; Sigal, 1993; Garcia-Monco et al., 1995). Immunodiagnosis and how this may be affected is another consideration. A whole-cell lysate vaccine would induce polyclonal antibody responses to multiple antigens that would make differentiation between vaccination and infection difficult. Similarly, conserved antigens amongst spirochetes and other bacteria could confound interpretation of diagnostic tests for Lyme (Shin et al., 1993). Subunit vaccines, rather, would induce responses to single or few antigens, easily distinguished from an infection response. Ancillary to this consideration is the question of how protection and efficacy can be determined, if a serological approach is required. For *Borrelia*, a pathogen with multiple species and variants found on several continents, concern about whether or not the vaccine will protect against other genospecies or variants should be included. Also, given the ability of the tick vector to harbor and transmit multiple pathogens concurrently upon feeding, the protection against possible co-infections must be taken into account. Lastly, and of significant importance, is the duration and type of immunity elicited. The generation of long-lasting B cell memory responses to *B. burgdorferi* or tick antigens would be ideal. This would limit the need for multiple booster injections to retain immunity.

## Bacterins and veterinary vaccines

Early studies on the immunogenicity of whole cell, killed (bacterin) preparations of the spirochetes demonstrated protection in hamsters w/formalin-inactivated *Borrelia* (Johnson et al., 1986a). Serum from vaccinated animals protected in passive immunization studies, indicating that protection is largely, if not completely, antibody-mediated (Johnson et al., 1986b). Not long after the discovery of the Lyme disease agent, natural infection of dogs became apparent (Lissman et al., 1984; Kornblatt et al., 1985; Magnarelli et al., 1985). As such, so did the interest in a veterinary vaccine. Currently, several licensed canine vaccines have become available. Bacterin vaccines, manufactured by Fort Dodge Labs (now Pfizer) (Chu et al., 1992; Levy et al., 1993) and Schering-Plough Animal Health (Galaxy Lyme) are available. Two subunit vaccines have also progressed to market—one consisting of just the *B. burgdorferi* outer surface protein A (OspA) subunit, Recombitek®, manufactured by Merial (Conlon et al., 2000), and another combining OspA and OspC subunits (Novibac® Lyme) by Merck Animal Health. These subunits will be discussed extensively in the next section. Though these vaccines appear to exhibit satisfactory efficacy, safety (side effects) and necessity continue to be under scrutiny (Littman et al., 2006). In certain circumstances serological surveillance of dogs can be used as a measure of endemicity for human Lyme disease (Rand et al., 1991; Hamer et al., 2009). Vaccination with the bacterin may interfere with this type of surveillance unless a properly chosen test (O'Connor et al., 2004) is used, so in that regard the subunit vaccines may be preferred.

## Ospa: the FDA-approved transmission-blocking vaccine

Several antigenic subunits of *B. burgdorferi* have been evaluated for their vaccine potential, many of which are listed in Table 1. For one reason or another, all antigens listed, other than OspA, have not been legitimized as vaccine candidates on their own. OspA is a lipoprotein whose expression is abundant on *in vitro*-cultured spirochetes and spirochetes within the tick midgut. This lipoprotein is also quite immunogenic, as antibodies are detected in experimentally infected animals. Importantly, due to the expression of *ospA* in a natural infection largely confined to the tick midgut, antibodies are not typically induced following tick-mediated infection. Compared to the immunodominant *B. burgdorferi* antigen, OspC, the OspA lipoprotein is reasonably well-conserved among North American strains; immunization with OspA, but not OspC, was shown to provide cross-protection of mice challenged with North American isolates of *Borrelia burgdorferi* (Probert et al., 1997). Sequence analysis revealed that the *ospA* genes from these three isolates were >99% homologous, whereas the *ospC* genes shared only 81–85% homology. Western blot analysis suggested antigenic heterogeneity associated with OspC but not OspA. The production of polyvalent chimeric OspC molecules may, however, enhance the potential for its use as an immunogen against Lyme disease (Earnhart and Marconi, 2007a,b; Earnhart et al., 2011).

**Table 1:** **Prospective Lyme vaccine antigens from *B. burgdorferi***.

***B. burgdorferi* antigen**	**Mechanism of protection**	**How tested**	**Result**	**References**
OspA	Antibody-mediated, transmission-blocking	Challenge of mice by injection, tissue transplant and tick transmission; challenge of monkeys by tick transmission	Efficacious, dependent upon antibody titer	Fikrig et al., 1990, 1992a,b; Probert and Lefebvre, 1994; Telford et al., 1995; Philipp et al., 1997
OspB	Antibody-mediated; elicits bactericidal antibodies	Active and passive protection against injection challenge	Potential for strain-dependent efficacy, due to truncations of OspB proteins in some strains	Fikrig et al., 1993; Telford et al., 1993; Coleman et al., 1994; Probert and Lefebvre, 1994; Probert et al., 1997
OspC	Antibody-mediated, within host	Challenge of mice by injection and tick transmission	Effective, but with minimal cross-species protection; failure to elicit long-term (anamnestic) response	Probert and Lefebvre, 1994; Gilmore et al., 1996, 2003; Probert et al., 1997
DbpA	Antibody-mediated, within host	Challenge of mice by injection and tick transmission	Protective against injected, but not tick-transmitted infection	Hanson et al., 1998; Hagman et al., 2000
Bbk32 (p35)	Antibody-mediated, within tick	Passive immunization against injection and tick challenge	Efficacy in combination with DbpA and OspC against challenge by injection but not singly	Fikrig et al., 1997, 2000; Brown et al., 2005

Initial studies on the protective efficacy of OspA utilized a mouse model of vaccination with recombinant OspA protein (Fikrig et al., 1990). Long-term (180 days) protection was elicited against challenge by intradermal inoculation of cultured *B. burgdorferi* (Fikrig et al., 1992a). Immunization in this manner was shown to be ineffective against challenge with host-adapted spirochetes, transferred by transplant of skin from an infected mouse to a naïve mouse (Telford et al., 1995). This finding, we now know, resulted from the aforementioned down-regulation of *ospA* expression once the spirochetes enter and adapt to the host. This variation in antigen expression compels the testing of any putative immunogen against *B. burgdorferi* challenge by tick-mediated infection. When immunization with OspA was tested in this manner, protective efficacy was found to be significant, and the spirochetes were eliminated from the tick vector by the serum antibodies post-feeding (Fikrig et al., 1992b). Eventually, the mechanism of protection as it related to expression of *ospA* by the spirochetes inside ticks was firmly established (De Silva et al., 1996). A subsequent study demonstrated that the anti-OspA antibody titer required to eradicate spirochetes from feeding ticks (>213 μg/ml) was considerably higher than that required (>6 μg/ml) for blocking transmission (De Silva et al., 1999).

These groundbreaking studies led to the eventual testing of safety, immunogenicity, and efficacy in the non-human primate model of *B. burgdorferi* infection (Philipp et al., 1997). Manufacture and distribution of the recombinant OspA vaccine, LYMErix, began by SmithKlineBeecham and it became available to the public in December 1998. LYMErix contained lipidized OspA adsorbed onto aluminum hydroxide adjuvant in phosphate-buffered saline with phenoxyethanol as a preservative (pH 6.5–7.1). LYMErix was produced using recombinant DNA technology. The OspA gene from the ZS7 strain of *B. burgdorferi* sensu stricto was placed in the pOA15 plasmid vector and grown in *Escherichia coli.* The OspA lipoprotein produced was a single polypeptide chain of 257 amino acids; the lipid moiety was covalently bonded to the N-terminus after translation. Each 0.5 ml dose containing 30 μg of lipidated OspA with adjuvant was administered intramuscularly, and three injections were recommended. LYMErix was available only through February 2002 and was not formulated in combination with other vaccines.

### The OspA vaccine's demise

Further evaluation of the immune responses of OspA-vaccinated animals led to the identification of a target (epitope) of specific antibodies that was strongly correlated with a protective response (Golde et al., 1997). The epitope was designated LA-2, after the monoclonal antibody originally used to identify it. Later, the importance of antibodies that targeted LA-2 in humans immunized with LYMErix also became apparent (Steere et al., 1998). Though the OspA vaccine emerged in an environment with Lyme disease reporting on the rise and heightened awareness of the risk, both geographically and health-related, it was eventually withdrawn from the market due to poor sales. Some of the pros and cons of the OspA vaccine can be found in Table 2. Regarding efficacy, the need to keep serum antibody titers high with multiple boosts, given the absence of antigen re-introduction at tick-transmitted infection, was suboptimal. However, the public response to the vaccine is likely what had the most influence on its diminished use. In particular, the proposed autoimmune potential for OspA due to its partial homology with the human lymphocyte function associated antigen-1 (hLFA-1) was brought into question. Patients with treatment-resistant Lyme arthritis were reported to possess specific HLA alleles, which retain the ability to present this autoantigen (Gross et al., 1998; Trollmo et al., 2001). Such HLA alleles, however, were not found more commonly in persons who developed arthritis after taking the OspA vaccine, calling its etiology into question (Ball et al., 2009). Less than 10% of vaccinated individuals reported side effects, including mild, local reactions and mild arthralgia (dose incidence 0.2%) from three different formulations of the vaccine; this result was not significantly different from the placebo group (Van Hoecke et al., 1996). Nonetheless, reports emerged suggesting that the vaccine could be arthritis-inducing (Rose et al., 2001; Lathrop et al., 2002). This led to anti-vaccine sentiment and class action lawsuits, along with reduced support amongst physicians for the vaccine and eventually enough of a decline in use for its voluntary removal by the manufacturer. Unfortunately, this failure in North America led the leading European prospective Lyme vaccine manufacturer, Pasteur Merieux Connaught, to halt development.

**Table 2:** **Positive and negative characteristics of the OspA vaccine**.

**PROs**	**CONs**
Blocks transmission; easier to test for efficacy	Required maintenance of high antibody titers for efficacy (multiple boosts)
Subunit; does not interfere with immunodiagnosis	Some adverse reactions, potential for inducing autoimmunity
Targets a reasonably conserved protein within species	Not effective against other tick-borne diseases

## Targeting the reservoir (mouse) host

Another option for interruption of transmission of *B. burgdorferi* to humans is through vaccination of reservoir hosts. In the North American regions of endemicity, *Peromyscus leucopus*, or the white-footed mouse, is considered the primary reservoir host, especially for larval and nymphal feeding (Levine et al., 1985; Anderson et al., 1987; Anderson, 1989; Mather et al., 1989). The majority of human infections are transmitted by Ixodid ticks in the nymphal stage (Barbour and Fish, 1993) so blocking acquisition at the pre-nymphal (larval) stage would be most effective for preventing human infection. Though such a task may seem daunting, the strategy remains viable for zoonotic infections with geographic “hot spots.” The control of Rabies virus in animal populations as a method to prevent human infection has proved effective (Niin et al., 2008; Sterner et al., 2009).

Considerations for a reservoir host vaccine include the antigen type and route of delivery, the type of delivery system, and the implementation protocol. For generating immunity against *B. burgdorferi* infection, antigens that target both the spirochetes (e.g., OspA) and the tick have been tested. Because the OspA antigen emerged as the most efficacious vaccine in animals and also acted by blocking transmission, it was the primary choice for the first reservoir-targeting vaccine strategies. Also, most of the studies that characterized OspA as a protective immunogen utilized mice, indicating that the outbred (wild) population would likely respond to vaccination with OspA. Initial studies were performed on plots with different densities of white-footed mice (Tsao et al., 2004). Using this catch and release strategy to vaccinate mice subcutaneously with OspA, the researchers found that vaccination did have an impact on the percentage of infected ticks the following year. This reduction in Borrelia-harboring ticks was positively correlated with the density of both ticks and mice, suggesting that targeting mouse-dense areas can have a significant impact on carriage, but the contribution of non-mouse species must also be considered.

Subsequent studies have focused on an approach that may be more practical on a larger scale, which is the baited oral vaccination strategy (Gomes-Solecki et al., 2006). Here, the antigen delivery method must be stable and effective by the oral route. Protection of mice (89%) and reduction of *B. burgdorferi* in vector ticks was accomplished by oral vaccination of the mice with *E. coli* expressing recombinant OspA (Gomes-Solecki et al., 2006). Advantages to baited oral vaccination of reservoir (mice) hosts with *E. coli* are the efficacy and the absence of safety issues, wherein the consumed vaccinogen is non-pathogenic and immunity does not wane over time, given a sufficient amount of antigen (Meirelles Richer et al., 2011). Another lead group using this strategy has chosen delivery of OspA by Vaccinia virus (VV) for several reasons: (1) these viruses have a broad host range; (2) VV are stable under harsh conditions, such as encountered in the digestive tract; (3) proteins can be expressed at high levels from VV and only a single dose is required; and (4) ingestion of VV does not cause disease in wildlife nor is it readily transmissible amongst infected animals (Bhattacharya et al., 2010). However, the potential to transmit the virus to unwanted recipients remains (CDC, 2009). Under laboratory conditions, C3H mice vaccinated by oral gavage with VV-OspA generated readily detectable antibody titers that peaked at 42 days post-inoculation (Scheckelhoff et al., 2006). Importantly, where 67% of ticks that fed upon control vaccinated mice were *B. burgdorferi*-positive, only 17% of those fed upon VV-OspA vaccinated mice harbored *B. burgdorferi*. The VV-OspA was later tested in a durable bait formulation with outbred *Peromyscus* mice to examine immune responses and the effects on tick transmission (Bhattacharya et al., 2011). Vaccinated mice developed antibody titers above the minimum required to prevent transmission, which waned slowly over time (30–40 weeks). In protection studies, 10/12 vaccinated mice were protected from infection (5 weeks p.i.) and the acquisition of *B. burgdorferi* by larval ticks fed upon infected mice was reduced from 85 to 23% with vaccination.

The tick protein, subolesin, has also been produced in a recombinant VV vector for use as a reservoir vaccine (Bensaci et al., 2012). This strategy holds the potential to prevent human infection with not just *B. burgdorferi*, but other pathogens such as Babesia and Anaplasma species that are often co-transmitted. The goal in this case is to prevent tick feeding and uptake of these pathogens from the reservoir hosts. Initial studies with this vaccine in laboratory mice demonstrated a significant reduction (52%) in tick feeding and a modest, albeit significant reduction (40%) in the transmission of *B. burgdorferi* by ticks to vaccinated mice. Perhaps a combination vaccine including OspA and subolesin would show enhanced efficacy.

The formulation consisting of the OspA antigen expressed by *E. coli* was incorporated into bait and tested on outbred *P. leucopus* mice. These studies showed that the majority of vaccinated mice generated antibodies to a key epitope (LA-2), were protected from tick challenge, and significantly reduced the *B. burgdorferi* prevalence in nymphs when infected ticks were fed upon vaccinated mice (Meirelles Richer et al., 2011). Using different vaccine dosing strategies, the authors demonstrated that by offering repeated dosages from 4 to 16 weeks, antibody titers could be kept high over a full year. The strategies discussed await field-testing, with some initial work having been performed (Telford et al., 2011). Nonetheless, by combining the knowledge gleaned from each of these studies, it is conceivable that reservoir-targeted vaccines could become a reality for implementation in coming years.

## Targeting the tick vector

Historically, vaccines against infectious agents, including *B. burgdorferi*, have primarily utilized live attenuated pathogens or antigens of the pathogen (Plotkin and Plotkin, 2011) to induce protective immunity. A potent alternative avenue to protect against arthropod-borne pathogens is targeting the vector itself, be it to eliminate the vector by using chemicals toxic to that vector (Carroll et al., 2009; Rosario-Cruz et al., 2009; Raghavendra et al., 2011), by para-transgenic approaches that modify the vectors' ability to transmit pathogens or reproduce (Aksoy et al., 2008; Hurwitz et al., 2011), or by use of vaccines targeting vector antigens critical for the vector to feed, reproduce or transmit pathogens (Wikel, 1988; De La Fuente et al., 2011; Mathias et al., 2012; Parizi et al., 2012). In this section of the review we will focus on the utility of vector-based vaccines against *Ixodes* species that transmit several human pathogens including *B. burgdorferi*, and provide a cohesive overview of some of the research efforts that might lead to a “next-generation” vaccine against *Ixodes* ticks and the pathogens they transmit in North America and Eurasia (Goodman et al., 2005).

### The hard life (cycle) of ixodes

A unique feature of the spirochetes of the *B. burgdorferi sensu lato* complex when compared with other pathogenic spirochetes is that it is entirely dependent on the obligate hematophagous Ixodes tick to infect susceptible vertebrate hosts (Piesman and Schwan, 2010). *B. burgdorferi* is transmitted by four species of Ixodes ticks within the *Ixodes ricinus* complex, *Ixodes scapularis* and *Ixodes pacificus* in the North America, and *Ixodes ricinus* and *Ixodes persulcatus* in Europe and Asia, respectively (Goodman et al., 2005). Additionally, *Ixodes scapularis* transmit *Anaplasma phagocytophilum*, *Babesia microti*, and Powassan virus in the North America and *I. ricinus* and *I. persulcatus* transmit tick-borne encephalitis virus in Europe and Asia (Kurtenbach et al., 2006). The ticks and the pathogens they transmit are maintained in a zoonotic cycle involving a diverse array of vertebrate hosts effectively increasing the breadth of the transmission cycles of the pathogens (Barbour and Fish, 1993; Keirans et al., 1996; Kurtenbach et al., 2006). We will focus on *I. scapularis*, the predominant vector of *B. burgdorferi*, *A. phagocytophilum*, *B. microti*, and Powassan virus in North America.

*Ixodes* spp have three developmental stages, the larvae, the nymph, and adult, and to complete development each stage requires one blood meal on a vertebrate host. In North eastern and North central America *Ixodes scapularis* larval and nymphal stages feed on small rodents such as *Peromyscus leucopus*, the predominant host (Barbour and Fish, 1993). Larvae hatch clean from eggs laid by mated female adults, acquire the pathogen/s from an infected host during feeding, and molt to become infected nymphs. When infected nymphs feed on mice, the pathogens are transmitted to the host. The nymphal stage is therefore central to pathogen transmission to reservoir hosts. *P. leucopus* is the major reservoir host for *B. burgdorferi* sensu stricto (Barbour and Fish, 1993). It is not clear if *P. leucopus* also serves as the predominant reservoir host for other *I. scapularis*-transmitted pathogens. While humans are not natural hosts for *Ixodes* ticks, ticks feed on humans upon accidental encounters of humans with infected nymphs and this often results in transmission of the pathogen/s (Kurtenbach et al., 2006). The larval acquisition of *B. burgdorferi* from the vertebrate host and subsequent molt to infected nymphs are thus critical determinants of infection prevalence, and consequent risk of infection to humans (Brunner et al., 2011). Infected nymphs molt to become infected adults. Adult *Ixodes* feed on white-tailed deer, but deer do not serve as reservoir hosts for *B. burgdorferi* (Nelson et al., 2000). It is suggested that components of deer serum, especially serum complement components, are Borrelicidal (Nelson et al., 2000). Further, larvae do not feed on deer, smaller mammals and rodents being their preferred hosts (James et al., 1990; Schmidt et al., 1999), additionally precluding the ability of deer to transmit *Borrelia* to larvae. Nymphal feeding precedes larval feeding, usually beginning in early spring and ending in late summer. This sets the stage for larvae to acquire pathogens that nymphs might have transmitted to the murine host. Larval feeding begins in late summer through fall and molt to become nymphs. Nymphs over-winter and begin feeding in the subsequent spring. Nymphs that fed in spring/summer molt to become adults and female adults begin feeding in late fall and winter. Fed adults lay eggs in early spring and larvae hatch in summer ready to acquire pathogens.

In the western United States, the life cycle of the western black-legged tick, *Ixodes pacificus* that is a vector for *B. burgdorferi* is complex and involves another *Ixodes* species, *I. spinipalpis. I. spinipalpis* larvae and nymphs feed on small rodents that serve as reservoir hosts for *B. burgdorferi*. *I. pacificus* larvae and nymphs routinely feed on lizards and lizards are incompetent reservoir hosts for *B. burgdorferi* (Lane and Quistad, 1998). Only occasional feeding of *I. pacificus* larvae and nymphs on *B. burgdorferi*-infected rodents results in infected *I. pacificus* stages competent to transmit the spirochete to humans (Peavey and Lane, 1995; Salkeld and Lane, 2010). Susceptible to changes in climate, ecology, and host population densities, the zoonotic life cycle of Ixodes ticks is thus closely entwined with the hosts it feeds on, and the pathogens it harbors (Kurtenbach et al., 2006).

### Vaccine targeting the tick—where do we begin?

In our pursuit for tick-based vaccines against *B. burgdorferi* in particular, and *I. scapularis* -transmitted pathogens in general, it is important to bear in mind that vaccines directed against tick stages that transmit pathogens are vital. Larvae rarely hatch infected (Patrican, 1997; Richter et al., 2012) and therefore are unable to transmit the currently known tick-borne pathogens. Infected nymphs are fully capable of transmitting harbored pathogens to humans, and domestic animals (Barbour and Fish, 1993). Further, the small size of the nymphal stage makes it difficult to notice and remove easily, hence can remain attached to the human host long enough to promote transmission of pathogen/s (Piesman et al., 1987). Infected female adults are likely to have higher pathogen burdens (having had twice the opportunity to acquire pathogens) and are fully capable of feeding and transmitting pathogens to humans. However the larger size of the female adult stage makes it easily noticeable and briskly removed, providing much less opportunity for transmission to ensue. Therefore, the general consensus is that targeting the nymphal stage might be most relevant from a human vaccine perspective. Efforts directed against larvae and larval acquisition of pathogens impact prevalence of infected nymphs in endemic areas, and efforts directed against adult ticks to increase their mortality and decrease fecundity impact tick population densities in endemic areas (Tsao et al., 2012). The vaccine target search cannot be compartmentalized; often proteins expressed in the nymphal stage are expressed in both larval and adult stages and proteins critical for feeding in one stage might also be critical for other stages (De La Fuente et al., 2011; Schuijt et al., 2011a; Bensaci et al., 2012). Tick salivary lectin pathway inhibitor protein (TSLPI) is critical for transmission of *Borrelia* to the murine host and also critical for *Borrelia* acquisition from the murine host (Schuijt et al., 2011a). Candidate vaccines that specifically target larval feeding or *Borrelia*/pathogen acquisition from the murine host naturally have to be delivered to the reservoir host, *P. leucopus.* So also vaccine candidates that specifically target adult tick feeding and fecundity have to be delivered to the reservoir host, *O. virginianus* (white-tailed deer). We will first focus the review on nymphal antigens, since vaccine candidates that target nymphal feeding and pathogen transmission have utility for reservoir host and humans.

### Low-hanging fruits—nymphal salivary proteins critical for feeding

*I. scapularis* nymphs feed for 3–5 days, gradually engorging to repletion and falling off the host. During this feeding process, the nymph inserts its hypostome into the host skin and tears the host skin with its sharp mouth parts (mandibles) and lodges itself firmly at the feeding site with a cement cone “glue” that the salivary glands secrete in preparation for feeding and pathogen transmission (Sonenshine, 1991). This event marks the onset of feeding and is critical, for it brings in close proximity the tick vector, its harbored pathogens, and the host dermis with all its inflammatory arsenals (Nuttall et al., 2000; Nuttall and Labuda, 2004; Hovius et al., 2008b). This event also initiates a series of molecular signals resulting in physiological changes in the tick gut and salivary glands critical for successful engorgement (Sauer and Hair, 1986). Success may be determined entirely by the ability of the tick to surmount host defense responses. The salivary glands secrete pharmacologically active molecules (Ribeiro and Francischetti, 2003; Nuttall and Labuda, 2004; Hovius et al., 2008b) that defuse host immune responses including host complement, pro-coagulants, proteases, histamine-binding proteins, and innate immune cells that are recruited to the feeding site. This prepares the ground for the exiting pathogens harbored by the tick, and facilitates pathogen transmission, a phenomenon described as Saliva Activated Transmission (Nuttall and Labuda, 2004). The *Ixodes* salivary transcriptome elaborates a variety of histamine binding proteins, anti-complement proteins, anti-coagulants, peroxidases, and protease inhibitors (Francischetti et al., 2005; Ribeiro et al., 2006), a handful of which have been fully characterized (Paesen et al., 1999; Valenzuela et al., 2000; Gillespie et al., 2001; Muleng et al., 2001; Francischetti et al., 2002, 2003, 2004; Narasimhan et al., 2002, 2007b; Sangamnatdej et al., 2002; Daix et al., 2007; Schroeder et al., 2007; Guo et al., 2009; Juncadella and Anguita, 2009; Schuijt et al., 2011a,b). Further, the genome encodes structural paralogs of several genes, whose functions remain to be determined (Hill and Wikel, 2005; Pagel Van Zee et al., 2007). Several questions arise: Why this structural paralogy? Are structural paralogs also functional paralogs? If so, are all the paralogs expressed simultaneously? Are the structural paralogs stage-specific? Structural paralogs are thought to represent a fall-back strategy, but this has not been fully tested. Because, ticks feed on multiple hosts, the structural and functional paralogs might be preferentially expressed on different hosts (Narasimhan, Unpublished) and might redirect our search for vaccine candidates tailored for targeting tick feeding on reservoir hosts or humans. The multifaceted strategies of the tick are the bane of research efforts to identify tick-based vaccine candidates. Nevertheless, since feeding is central to tick survival and pathogen transmission, targeting salivary proteins critical for feeding presents a logical starting point. Further, salivary proteins are secreted into the host at the feeding site and have the added advantage of providing anamnestic responses to boost vaccine efficacy.

A few salivary proteins have been tested for their vaccine potential to block nymphal or adult feeding, with some promise (Hovius et al., 2008b; Parizi et al., 2012). While many provide impaired feeding, blocking tick feeding has been difficult. Clearly, ticks encode an impressive array of molecules to defuse host immune responses and vaccines targeting one or two components are not sufficient to derail tick feeding effectively. For example, *I. scapularis* salivary protein molecules such as ISAC (Valenzuela et al., 2000), Salp20 (Tyson et al., 2007), Salp15 (Schuijt et al., 2008), and TSLPI (Schuijt et al., 2011a) inhibit different arms of host complement pathways and vaccines that target these in combination might be a more viable option; this has yet to be tested. Similarly, anticoagulant proteins including Ixolaris, Penthalaris, Salp14 (Francischetti et al., 2002, 2004; Narasimhan et al., 2002), and P28 (Schuijt et al., 2011b), identified from *I. scapularis* adult or nymphal salivary glands inhibit different components of the coagulation cascade and might be targeted in combination rather than individually to inhibit successful feeding. More recently, *I. ricinus* salivary glands were shown to elaborate a novel Serpin, IRS-2, that defused host inflammation and thrombin-induced platelet aggregation (Chmelar et al., 2011). Homologs of *I. scapularis* IRS-2 might be added to a vaccine cocktail targeting several anticoagulants to block tick feeding. Mining the genome and transcriptome reveals a long list of putative secreted proteins that might be vaccine targets, but the daunting task of prioritizing a set of potential targets remains the confounding obstacle.

### Acquired resistance to ticks—just a matter of time

**Figure 2:**
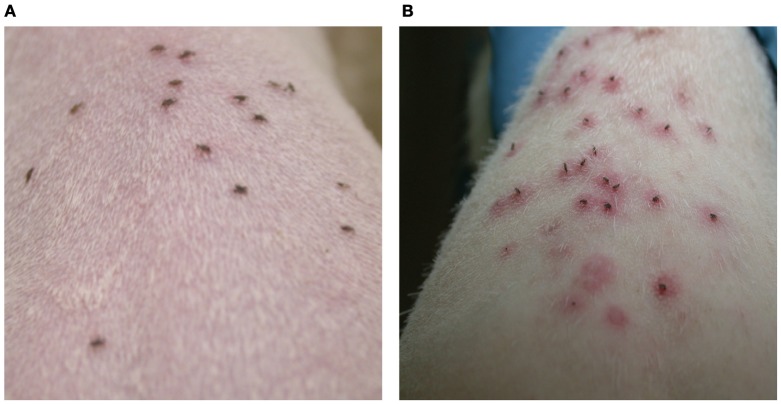
***Ixodes scapularis* infestations of guinea pigs results in the development of acquired resistance to ticks.** Nymphs feeding on: **(A)** naïve guinea pig shows no redness at the tick bite-sites and **(B)** repeatedly tick-infested guinea pig shows increased redness around the tick bite-site within 24 h of tick attachment.

Several decades ago, William Trager observed that rabbits infested repeatedly with *Dermacentor* ticks develop a robust immune response against tick components that results in rapid rejection of ticks (Trager, 1939), and since then this phenomenon of acquired tick resistance has been noted in various tick-host models (Wikel and Alarcon-Chaidez, 2001). *I scapularis* ticks feed successfully on guinea pigs and rabbits, the laboratory models of non-reservoir hosts, at first infestation, but subsequent infestations result in dramatic reduction in feeding and ticks fall off or die within 12–24 h (Allen, 1989). Interestingly, this phenomenon does not occur upon repeated infestations of *I. scapularis* ticks on the murine host, the chosen reservoir host (Wikel et al., 1997), for reasons that are not well understood. The hallmark of tick resistance is the swelling and redness at the tick bite-site (Figure 2) due to cutaneous basophil hypersensitivity, or the rapid recruitment of basophils to the tick bite-site (Brossard and Fivaz, 1982; Wikel and Alarcon-Chaidez, 2001) and is apparently mediated by the concerted activation of humoral and cellular responses. Recruitment of basophils to the bite site, followed by their degranulation, effectively thwarts tick feeding, and promotes tick mortality by mechanisms that are not fully understood (Brown, 1982; Brown and Askenase, 1983, 1985). It is presumed that salivary proteins secreted into the bite site provoke the immune response in the host that recruits basophils to the site (Brown and Askenase, 1985; Wikel and Alarcon-Chaidez, 2001). Importantly, when *B. burgdorferi*-infected nymphs were allowed to feed on tick-immune guinea pigs, *B. burgdorferi* transmission was also dramatically impaired (Nazario et al., 1998; Narasimhan et al., 2007a). Hence, there is an ongoing interest to exploit the phenomenon of acquired tick resistance to identify tick salivary proteins that are natural targets of host immunity (Schuijt et al., 2011b). It is anticipated that this would help define salivary protein candidates that might serve as vaccine targets to block tick feeding and *Borrelia* transmission.

Different molecular approaches have been utilized to identify proteins in engorged tick salivary glands that react with tick-immune sera and several *I. scapularis* nymphal salivary proteins with interesting biochemical functions have been identified (Das et al., 2001; Schuijt et al., 2011b). Despite the robust reactivity of these proteins with tick-immune sera, that include peroxiredoxins, anticoagulants, anticomplements, and histamine binding proteins, the vaccine potential of these proteins when tested in a cocktail to block nymphal and adult tick feeding remains modest (~20–30% reduction in engorgement weights) (Schuijt et al., 2011b). Further, not all ticks in the immunized group are affected, and this variation in efficacy also poses a bottleneck, especially from a human vaccine perspective.

**Figure 3:**
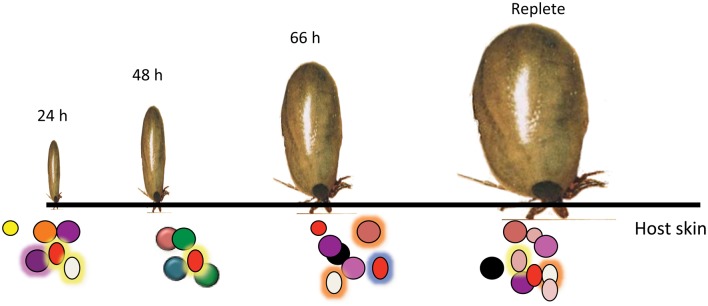
**Temporally changing composition of tick saliva spit into the host skin.** Schematic representation of the dynamic tick saliva. *Ixodes scapularis* engorge on vertebrate host skin for 3–7 days spitting saliva into the host dermis at the bite-site. Salivary composition potentially changes during feeding to confront the different host defense responses.

A careful analysis of the tick salivary transcriptome suggested that the tick salivary proteome might be dynamic, and change during feeding (Narasimhan et al., 2007a). Feeding proceeds not as one “big gulp” nor as a steady “sipping,” but proceeds in phases defined grossly as slow in the first 1–2 days and then rapid in the last 3rd and 4th day (Anderson and Magnarelli, 2008). It is then plausible that the salivary proteome changes to meet feeding phase-specific requirements (Figure 3). Histopathological and molecular examination of the dermis at the tick bite site also showed differences in the composition of the inflammatory milieu that accumulates in the early and late stages of feeding (Krause et al., 2009; Heinze et al., 2012). During the final rapid feeding phase, *I. scapularis* ticks have been shown to secrete a protein that facilitates release of histamines from neutrophils, mast cells and possibly basophils to increase vasodilation and accelerate the flow of blood to the bite site (Dai et al., 2010). Understanding the dynamics of the tick proteome reveals a possible drawback in the approach to identification of tick salivary proteins targeted by host tick-immunity. Since acquired resistance to tick feeding results in rapid tick rejection within the first 12–24 h of tick attachment, presumably, host immunity is directed against salivary proteins expressed in the early phase and it might be critical to identify this subset of salivary proteins. Perhaps we have to shift the focus away from antigens expressed later in feeding, to antigens expressed early in tick feeding. Targeting salivary proteins expressed early in feeding has the advantage of blocking tick feeding early and blocking the transmission of pathogens such as TBEV, *A. phagocytophilum* and *B. microti* that are transmitted earlier in feeding than *B. burgdorferi* (Goodman et al., 2005). The process to dissect and obtain sufficient amount of proteins or RNA from 12 to 24 h fed salivary glands is tedious. Yet, powerful molecular approaches are now available to circumvent the limitations of this approach and to spur the analysis of the early phase transcriptome and proteome of *I. scapularis* (Hill and Wikel, 2005).

### Blocking *borrelia* transmission—the real deal

Blocking tick feeding might just be a monumental task, up against the powerful evolutionary measures designed to ensure that the tick saliva is equipped with protein and non-protein biomolecules critical for feeding (Oliveira et al., 2011). But, from a human vaccine perspective, do we really want to block tick feeding? Is it sufficient that we block pathogen transmission? A vaccine that can effectively block pathogen transmission is undoubtedly the public health goal and broadly applicable to the murine reservoir host and to humans. The first transmission blocking vaccine against *B. burgdorferi*, based on the *Borrelia* outer surface protein OspA (Fikrig et al., 1992a,b), was developed in 1998, but removed from the market in 2001 due to the perceived notion that anti-OspA antibodies were inherently arthritogenic (Rose et al., 2001; Lathrop et al., 2002) as detailed in section “OspA: The FDA-Approved Transmission-Blocking Vaccine.” Therefore, a safe and effective vaccine against Lyme disease remains an unmet need.

64TRP, a subunit of a secreted salivary cement protein of *Rhipicephalus appendiculatus*, was tested in a guinea pig model of feeding and shown to provide a significant impairment of adult *R. appendiculatus* feeding (about 23–25%) and a ~45% reduction in egg mass (Trimnell et al., 2005). Further, since 64TRP shared structural homology with proteins from several tick species, including, *I. scapularis*, it has the potential utility of being a broad spectrum vaccine to target multiple tick species and consequently impair the transmission of multiple tick-transmitted pathogens. Indeed when 64TRP was tested in murine model of *I. ricinus* transmitted TBEV, the inflammation elicited at the feeding site in 64TRP-immunized mice impaired TBEV transmission from infected mice to nymphs, and from infected nymphs to mice (Labuda et al., 2006). This finding provides new optimism in the search for tick-based vaccines (Trimnell et al., 2005).

Two *I. scapularis* salivary cysteine protease inhibitors, Sialostatin L and Sialostatin L2, were shown to play a role in nymphal tick feeding (Schwarz et al., 2012). Inhibition of Cathepsin S by Sialostatin L decreased inflammation and possibly facilitated feeding (Kotsyfakis et al., 2008). Sialostatin L2 is speculated to have a role in modulating inflammatory responses, tissue remodeling and angiogenesis by inhibiting intra or extracellular cathepsins of innate immune cells (Kotsyfakis et al., 2010). More importantly, inoculation of mice with *B. burgdorferi* in conjunction with Sialostatin L2 significantly promoted spirochete survival and infection in the skin (Kotsyfakis et al., 2010), suggesting that a vaccine targeting Sialostatin L2 might help thwart *B. burgdorferi* transmission.

*I. scapularis* salivary protein, TSLPI, is an anti-complement protein that inhibits the lectin pathway of the complement cascade by binding to the mannose binding lectin (MBL) and preventing its ability to activate the downstream signaling (Schuijt et al., 2011a). When tested as a vaccine to block tick feeding, it was not effective (Schuijt et al., 2011b). *B. burgdorferi s.l* strains are sensitive to human complement (Van Dam et al., 1997), and *in vitro* studies on *B. burgdorferi s.l* strains showed that TSLPI offered protection from human complement, and antibodies against TSLPI abrogated the protective effect (Schuijt et al., 2011a). Both immunization against TSLPI and RNA interference-mediated decrease in TSLPI expression in *I. scapularis* nymphs impaired *B. burgdorferi* transmission by *I. scapularis* nymphs (Schuijt et al., 2011a). *I. ricinus* ticks also elaborate potent anticomplement proteins, IRAC I and II, that inhibit the alternative pathway of mammalian complement (Schroeder et al., 2007) and bear structural and functional homology to ISAC, the *I. scapularis* anticomplement (Valenzuela et al., 2000). It is then possible that cocktail vaccines of potent structurally related anticomplement proteins might serve as a broad-spectrum vaccine to prevent *B. burgdorferi* transmission by *I. scapularis* and *I. ricinus* ticks. Various proteins with anti-inflammatory functions have been characterized to-date from *Ixodes* species (Hovius et al., 2008b). Theoretically, it should be possible to test the vaccine potential of these proteins in tick challenge experiments in the context of *Borrelia* transmission as well as in the context of other pathogens transmitted by *I. scapularis* or *I. ricinus*. Testing them as a cocktail of functionally related proteins might be a more tractable approach to accelerate prioritization.

The discovery that Subolesin, a transcription factor expressed by several tick species including *I. scapularis*, can be targeted to decrease the feeding and fecundity of adult ticks, and feeding of larval and nymphal ticks has revealed a new facet to tick-based vaccine development (De La Fuente et al., 2011). Subolesin is a homolog of vertebrate akirins and is evolutionarily conserved in insects and in ticks (Galindo et al., 2009). As discussed in section “The OspA Vaccine's Demise,” oral vaccination of murine hosts with vaccinia viruses that express Subolesin provided protection against tick infestations and *B. burgdorferi* transmission (Bensaci et al., 2012). This could have utility as a reservoir-host vaccine. Importantly, like 64TRP, immunity to Subolesin effectively impaired the feeding ability of several tick species (De La Fuente et al., 2006; Merino et al., 2011). Since Subolesin homologs were also expressed in mosquitoes, it has the potential of being part of a broad-spectrum vaccine formulation (Canales et al., 2009). Subolesin is an intracellular protein that functions to transcriptionally regulate NF-kB-dependent genes (Galindo et al., 2009). The traditional approach is to target secreted salivary proteins or at least extracellular tick proteins. The mechanisms by which anti-Subolesin antibodies are able to enter tick cells to target or neutralize Subolesin is not fully understood, and opens new possibilities that intracellular tick proteins can also be targets for protective vaccines (De La Fuente et al., 2011).

### Separate and conquer—a new paradigm

It was shown a few years ago that TROSPA, a tick gut protein helps tether *B. burgdorferi* to the gut by binding to OspA, an outer surface protein of the spirochete, and that this facilitated spirochete colonization (Pal et al., 2004). When TROSPA expression was decreased in *I. scapularis* nymphs, *Borrelia* acquisition was impaired (Pal et al., 2004). Similarly, spirochetes that did not express OspA were unable to colonize the tick gut (Yang et al., 2004) and emphasized that molecular interactions between the tick and the spirochete were specific, and thus could be targeted to derail the ability of the spirochete to colonize the tick. Underscoring this understanding of spirochete-tick interactions, Salp15, a multifunctional secreted *I. scapularis* salivary protein has been shown to inhibit activation of CD4^+^T cells (Anguita et al., 2002), complement activity (Schuijt et al., 2008), and dendritic cell function (Hovius et al., 2008a). Salp15 was also shown to physically bind to OspC on the spirochete surface during exit from the salivary glands (Ramamoorthi et al., 2005; Rosa, 2005). Tilly et al. showed that OspC, an outer surface lipoprotein that decorates the spirochete surface as it exits the tick, is critical for *Borrelia*, early in infection of the vertebrate host (Tilly et al., 2006). It was suggested that Salp15-OspC interaction potentially cloaked OspC from host immune responses and protected the spirochete from *Borrelicidal* antibodies (Ramamoorthi et al., 2005). This provided a significant survival edge upon entry into the host, escaping the inflammatory host responses that accumulate at the tick bite site during feeding. Mice actively immunized with recombinant Salp15, and challenged with *B. burgdorferi*-infected nymphs were significantly protected from infection (Dai et al., 2009). It is likely that antibodies directed against Salp15 sequester Salp15 away from OspC and leave it exposed to the immune milieu, or Salp15 antibodies bind to Salp15-coated spirochetes and deliver the spirochetes more effectively to phagocytes (Dai et al., 2009). Salp15 homologs were also identified in *I. ricinus* ticks and similarly bound *B. garinii* and *B. afzelii* OspC to facilitate spirochete transmission (Hovius et al., 2008c).

During transmission, the spirochete replicates, and migrates from the gut to the salivary glands (Rosa et al., 2005), to then exit the vector and enter the host. Elegant live imaging of ticks infected with GFP-expressing spirochetes showed that *B. burgdorferi* associate tightly with the tick gut epithelial cells and move as a meshed network toward the basement membrane of the gut in preparation for egress from the gut (Dunham-Ems et al., 2009). Extending the concept of PIP, or Pathogen-Interacting-Proteins, to the tick gut, it has been shown that the *Borrelia* outer surface protein BBE31 binds to a gut protein, TRE31 (Zhang et al., 2011) to enhance its egress from the gut by mechanisms that remain to be elucidated. Disrupting the interaction between BBE31 and TRE31 compromised spirochete egress from the gut. These findings provide a new understanding of vector–pathogen co-operation that involves a direct interaction of the vector protein with the pathogen. These PIPs might be targeted as vaccines to prevent Lyme disease.

### The tick gut-targeting acquisition and transmission

Although the search for immunogens to block tick feeding and pathogen transmission is predominantly saliva-centric, the gut must not be ignored, for it is the entry and exit avenue for tick-borne pathogens. The blood-meal, critical for tick development, arrives in the gut and is stored in the gut for several days post-feeding as digestion proceeds (Sauer and Hair, 1986). The gut also presents a unique vector–host–pathogen interface wherein the vector secretes proteases, anticomplements, antibacterial peptides and anticoagulants to protect the epithelial barrier from pathogen and host-mediated damage and to keep the blood-meal fluid during the long feeding period (Rudenko et al., 2005; Anderson et al., 2008). Targeting gut components critical for the vector to continue feeding is an equally viable option (Nuttall et al., 2006). Salp25D, a peroxiredoxin expressed both in the salivary glands and guts was shown to be critical for *Borrelia* acquisition (Narasimhan et al., 2007b) by neutralizing reactive oxygen species at the vector–host interface and facilitating the viability of spirochetes as they entered the gut. It is conceivable that targeting TROSPA, and Salp25D simultaneously might help to effectively block *Borrelia* acquisition from murine hosts, and such a vaccination strategy could be applicable to reservoir host to impact infection prevalence. The gut also presents a daunting physical and immunocompetent entry-point barrier for tick-borne pathogens (Kopacek et al., 2010). Little is known about the molecular mechanisms that *Borrelia* and other pathogens utilize to surmount the barrier and an understanding of this should open new ways to block pathogen acquisition and transmission.

## Conclusions

Several tick molecules with the potential to serve as vaccines to impair feeding and transmission have been identified in the last decade. The sequencing of the genome of *Ixodes scapularis* (Hill and Wikel, 2005) has contributed largely to this progress and the time is ripe to put our collective efforts to develop an effective vaccine against Lyme disease. A tick-based vaccine holds the promise that it might be useful to also simultaneously block the transmission of other tick-borne pathogens (Wikel, 1996). The application of RNA interference technology to the tick field has catalyzed our ability to designate physiological functions to tick genes and to partially remove some of the bottle-necks that the field had faced a decade ago (De La Fuente et al., 2005; Karim et al., 2010). Technologies to genetically manipulate *I. scapularis* are also coming of age (Kurtti et al., 2008) and represent another milestone that will help increase our understanding of tick genes in the context of development, feeding, and pathogen transmission; this will help us prioritize tick antigens for vaccine development. Recent work has shown that immunization of murine hosts with a combination of Salp15 and OspA provided better protection from *B. burgdorferi* infection than either alone (Dai et al., 2009). Incorporating antigenic epitopes of critical tick and *Borrelia* proteins into a chimeric vaccine might thus be a viable option for Lyme vaccine development. Vaccination of the reservoir hosts and/or humans are not mutually exclusive options, and targeting the reservoir populations to decrease tick populations and interrupting acquisition or transmission cycles in conjunction with vaccination of humans should provide the desired goal of controlling tick-borne pathogens (Tsao et al., 2012).

### Conflict of interest statement

The authors declare that the research was conducted in the absence of any commercial or financial relationships that could be construed as a potential conflict of interest.
